# Treatment of Hospital Phagedæna

**Published:** 1855-04

**Authors:** 


### SHORT NOTICES OF HOSPITAL THERAPEUTICS.

*Treatment of Hospital Phagedæna.*—The prevalence of phagedæna, which, during the past nine months, has been pretty general in the London Hospitals, seems now to be steadily diminishing. It has been very difficult to arrive at any satisfactory conclusions respecting the laws regulating its occurrence. At one period, it has appeared to spread through a certain ward, or to prevail in a certain Hospital, as if by contagion; while, at others, observations have been made tending strongly to support the opinion, that it was largely under the influence of atmospheric changes. It has prevailed very irregularly in the different Hospitals, being now epidemic in one, and then, after the lapse of a short time, appearing in another. On the whole, it has been a mild form of the disease. Very few have, we believe, died of it and a vast majority have recovered after a short and not very destructive attack. In several instances, however, in which stumps, after amputation, have been attacked by it, so much of the soft parts have been destroyed, that a second removal of the bones has become necessary. With regard to the treatment of the disease, the following recommendations might, we think, be summed up as the results of the combined experience of the Surgeons who have been most engaged with it :—

1. *As soon as a wound shows a tendency to become sloughy or phagedænic, to have the patient change his bed, and if possible, his ward.*—This practice was pursued in almost all cases in Guy's and in the London Hospital, and more or less in most others. Often very sudden benefit was remarked. The recommendation, of course, proceeds on the supposed desirability of removing the patient away from any local influences, contagious or endemic, which may have had part in producing the disease. The following case may be quoted as illustrative :—A boy, in excellent health, submitted to primary amputation of the arm, on account of a crush, under the care of Mr. Wordsworth, in the London Hospital. On the day following the operation he was remarkably well, and had not the least constitutional disturbance. During the next six days he continued well, and the stump was granulating healthily, when it became necessary to change his bed, and to put him into one from which a man who had died of pyæmia had been removed. Mr. Wordsworth directed that all the bed furniture should, as a measure of precaution, be removed; and, with the exception of the mattrass, this order was complied with. On the morning following the change of bed, the lad was feverish and restless, and his stump had lost its granulations and presented a sloughy surface. He was at once ordered back to his original bed. The phagedæna did not spread; but, almost immediately after the second change, the condition of the stump began to improve, and ever afterwards the advance was uninterrupted.

2. *To destroy fetor by the employment of charcoal.* In this way, probably, not only is a gas decomposed which was likely to have acted prejudicially on the animal functions, but one which might not improbably have been the means of direct infection.

3. *To employ nitric acid as an application to the sore.* Most Surgeons have formed a very high opinion of the value of this remedy when efficiently used. The acid should be concentrated and pure, and should be liberally applied. We have already at such length spoken in its praise that anything further need scarcely be here added. (See *Medical Times and Gazette* for Jan. 6, page 9.)

4. *To employ as constitutional measures tonics and general stimulants with in some cases opium, or the chlorate of potash as specific remedies.* Respecting the use of the latter, a considerable difference of opinion prevails; but instances have occurred in some Hospitals which appeared to show almost incontestably their potency in at least some individual cases. The chlorate of potash well deserves a much more thorough investigation as to its remedial powers than it has yet received.

### *Stomachic Pill.*

The following is the prescription for an excellent dinner pill. We copy it from the Pharmacopœia of Guy's Hospital:—

Take of powdered capsicum, ʒi.; powdered rhubarb, ʒij. Make into a mass with treacle, and divide into sixty pills. Two or three to be taken every day before dinner.

### *Lotion of the Chlorate of Potash.*

In the Middlesex Hospital, Mr. Moore, and also several of his colleagues at his suggestion, have been largely using a lotion of the chlorate of potash as a dressing for unhealthy sores. It is considered to have been very successful. The strength has been from two to three drachms to the pint of water. It has been freely applied on lint.—*Medical Times and Gazette.*

